# Inflammatory Mammary Carcinoma in a Captive Bengal Tiger (*Panthera tigris tigris*) with Lymph Node and Pulmonary Metastases

**DOI:** 10.3390/ani16050757

**Published:** 2026-03-01

**Authors:** Ju-Won Kang, Jaewoo Choi, Hajin Jeong, Hyeon Jeong Moon, Gun Lee, Chung-Do Lee, Ho-Jin Lee, Min-Seop Song, Ji-Hyeon Kim, Yeong-Hun Ko, Hyunwoo Kim, Changmin Sung, Jun-Gyu Park, Yeong-Bin Baek, Sang-Ik Park

**Affiliations:** 1 Veterinary Medical Team, Gwangju Uchi Zoo, Gwangju 61028, Republic of Korea; cobe12@daum.net (J.-W.K.); gkwls1124@korea.kr (H.J.); khalls@korea.kr (C.S.); 2 Department of Veterinary Pathology, College of Veterinary Medicine, Chonnam National University, Gwangju 61186, Republic of Korea; e05311cht@naver.com (J.C.); dals404@naver.com (H.J.M.); dlghwls2458@naver.com (H.-J.L.); mrsongms1@gmail.com (M.-S.S.); kimjihyeon2017@naver.com (J.-H.K.); rhdudgns23@naver.com (Y.-H.K.); 3 Department of Veterinary Pathology, College of Veterinary Medicine and BK21 FOUR Program, Chonnam National University, Gwangju 61186, Republic of Korea; udlrjs77@naver.com (G.L.); cndehsla@gmail.com (C.-D.L.); 4 Noah Animal Medical Center, Gwangju 61426, Republic of Korea; hyunu0222@naver.com; 5 Department of Veterinary Zoonotic Diseases, College of Veterinary Medicine, Chonnam National University, Gwangju 61186, Republic of Korea; kingsalt@jnu.ac.kr

**Keywords:** dermal lymphatic tumor emboli, cribriform carcinoma, immunophenotype, pulmonary metastasis, lymph node metastasis, zoological medicine, captive felid neoplasia

## Abstract

Inflammatory mammary carcinoma (IMC) is an aggressive mammary carcinoma phenotype characterized by tumor emboli within superficial dermal lymphatic vessels and early metastasis. A captive Bengal tiger (*Panthera tigris tigris*) presented with large abdominal mammary masses and regional lymphadenopathy; contrast-enhanced computed tomography also revealed a pulmonary nodule. Postmortem examination and histopathology confirmed mammary carcinoma with dermal lymphatic tumor emboli and metastases to regional lymph nodes and the lung. Tumor emboli were cytokeratin positive, supporting epithelial origin and an IMC diagnosis, and neoplastic cells were immunopositive for cytokeratin with concurrent vimentin immunoreactivity. This case highlights the clinicopathologic basis of IMC and the diagnostic importance of including full-thickness skin and adjacent subcutis in the sampling plan.

## Simple Summary

Inflammatory mammary carcinoma (IMC) is a rapidly progressive mammary cancer in which tumor cells spread through superficial skin lymphatic vessels, leading to marked skin inflammation and early metastasis. We describe IMC in a captive Bengal tiger (*Panthera tigris tigris*) that showed large abdominal mammary masses, regional lymph node enlargement, and a pulmonary nodule on computed tomography. Necropsy and histopathology identified an invasive cribriform mammary carcinoma with severe tumor-associated inflammation and extension toward the superficial dermis. Tumor emboli were present within dermal lymphatic vessels and were confirmed as epithelial by cytokeratin immunostaining. Metastatic carcinoma was detected in regional lymph nodes and the lung, and neoplastic cells coexpressed cytokeratin and vimentin. This case emphasizes that IMC confirmation is sampling dependent and requires intentional submission of full-thickness skin and adjacent subcutis to evaluate dermal lymphatics.

## 1. Introduction

Mammary tumors are among the most common neoplasms in cats and are predominantly malignant carcinomas, with a propensity for early regional and distant metastasis and generally poor clinical outcomes [1,2,3]. Prognostic interpretation relies primarily on clinicopathologic stage and key pathologic variables, including histologic grade and lymphovascular invasion (LVI) [1,4,5,6]. In parallel, advances in breast cancer diagnostic technologies—including emerging optical and digital approaches—continue to inform translational diagnostic concepts and tool development relevant to mammary carcinoma investigations [7].

In managed-care settings, mammary neoplasia has become an increasingly recognized diagnostic and welfare concern in captive nondomestic felids [8]. A growing body of zoo and wildlife pathology literature suggests that neoplasia detection is higher in captive nondomestic felids under managed care than in free-ranging counterparts, plausibly reflecting longer life expectancy, improved surveillance, and enhanced access to diagnostic and necropsy evaluation [6]. These observations pertain to managed-care wildlife populations and should be interpreted separately from domestic-cat mammary tumor epidemiology. Within *Panthera* spp. under managed care, reproductive tract neoplasms are prominent, and mammary carcinomas constitute a substantial proportion of these lesions, often demonstrating widespread metastasis [9]. Moreover, retrospective and comparative analyses in zoo felids have repeatedly raised concern regarding exogenous progestin-based contraception (notably melengestrol acetate, MGA) as a potential risk factor for mammary carcinoma development, warranting careful documentation of reproductive management history in case investigations [10].

Among malignant mammary entities, inflammatory mammary carcinoma (IMC) is clinically critical because of its rapid progression, severe local disease, and high metastatic propensity [11]. Importantly, in IMC the term “inflammatory” primarily denotes a clinical syndrome (diffuse mammary/cutaneous erythema and edema resembling inflammation), rather than a diagnosis based on the presence of histologic inflammation alone. A recurring challenge in veterinary oncology and diagnostic pathology is that the label “inflammatory” is fundamentally a clinical descriptor; therefore, a defensible diagnosis requires a transparent clinicopathologic framework rather than reliance on secondary inflammation, ulceration, or nonspecific inflammatory infiltrates alone [12]. Consistent with current concepts of carcinoma progression and aggressiveness, oncogenic signaling programs implicated in invasion and metastatic competence provide a general mechanistic framework for aggressive phenotypes [13]. In IMC, however, the term “inflammatory” remains primarily clinical; therefore, objective clinicopathologic criteria—most importantly dermal lymphatic tumor emboli—are required to avoid conflating secondary inflammation with the IMC phenotype [14]. Clinically, IMC is typically associated with an acute-onset, rapidly progressive inflammatory phenotype affecting mammary tissue and overlying skin (e.g., erythema, edema, warmth, pain, firm induration, and rapid extension) [14]. Pathologically, the key confirmatory feature is prominent lymphovascular involvement—most characteristically, neoplastic emboli within superficial dermal lymphatic vessels of the overlying skin (“dermal lymphatic tumor emboli”), which provides an objective correlate for the inflammatory presentation [14].

Because this hallmark may be missed if sampling is limited to the mammary mass itself, methodologically robust investigations should explicitly include full-thickness skin and superficial dermis overlying/adjacent to the mammary lesion, enabling direct assessment of dermal lymphatics and supporting reproducible diagnostic interpretation.

Immunohistochemistry can provide additional biologic context for aggressive mammary carcinomas by confirming epithelial lineage (e.g., cytokeratins) and by assessing phenotypic plasticity programs associated with epithelial–mesenchymal transition (EMT) that have been linked to invasion and metastatic competence in feline mammary cancer [15]. Mechanistic studies in breast cancer further suggest that metabolic reprogramming can support tumor progression; for example, MAEL has been shown to promote aerobic glycolysis and malignant behaviors by facilitating chaperone-mediated autophagy-dependent lysosomal degradation of Krebs cycle enzymes (citrate synthase and fumarate hydratase) [16]. In cats, multiple studies have examined EMT-related marker patterns and their relationship with malignant behavior, supporting the rationale for documenting cytokeratin profiles and mesenchymal marker expression (including vimentin) when tumors display highly invasive growth, extensive LVI, or rapid metastatic dissemination [17,18,19]. Notably, vimentin expression in carcinoma cells is not specific for EMT; therefore, interpretation should be integrated with morphology, invasion patterns, and clinicopathologic behavior rather than be inferred from a single marker alone [15,17,18].

Here, we describe a mammary carcinoma with an inflammatory phenotype in a captive Bengal tiger (*Panthera tigris tigris*). We hypothesized that an IMC designation in a nondomestic felid requires integration of the rapidly progressive inflammatory clinical syndrome with objective confirmation of dermal lymphatic tumor emboli, which is best achieved through intentional full-thickness skin/subcutis sampling. Accordingly, the aim of this case report was to present a concise, pathology-centered diagnostic workflow—integrating clinical assessment and imaging with standardized necropsy sampling, histopathology, and immunohistochemistry—to provide a transparent clinicopathologic rationale for applying the IMC designation in a captive tiger.

## 2. Case Description

A 14-year-old captive female Bengal tiger housed at a zoological facility in the Republic of Korea was evaluated for progressive mammary masses with systemic deterioration manifested by lethargy, anorexia, progressive weight loss, and severe watery diarrhea with melena and suspected metastatic diseases, showing overlying cutaneous ulceration noted on clinical examination at the time of advanced evaluation. The clinical course was characterized by a rapidly progressive inflammatory mammary phenotype, progressing over approximately four months (January–May 2024) from initial detection of a focal mammary abnormality to extensive mammary masses with diffuse mammary and cutaneous changes (erythema, edema, warmth, pain, firm induration, and rapid extension of the affected region).

In January 2024, a small abnormal structure was first detected in the right caudal abdominal mammary region during routine monitoring. The lesion was initially observed without intervention. By May 2024, the animal developed severe watery diarrhea with melena and marked weight loss, prompting further diagnostic evaluation. In the interval between initial detection and the advanced evaluation, the mammary lesion showed clinically apparent acceleration in progression, with rapid expansion and development of diffuse mammary and cutaneous inflammatory changes in the weeks leading up to May 2024. Supportive management, including nutritional support, systemic antimicrobial therapy, and analgesia, was administered for approximately 7 days prior to advanced diagnostic work-up; despite this supportive care, the animal showed no appreciable improvement of gastrointestinal signs and overall systemic condition, leading to escalation to comprehensive diagnostic imaging and staging. Non-steroidal anti-inflammatory drugs (NSAIDs) were not administered given the presence of melena and concern for gastrointestinal bleeding, and systemic corticosteroids were not administered due to concern for potential secondary infection and ulceration associated with the mammary lesion; therefore, anti-inflammatory management remained supportive and risk-adapted in this clinical context.

Complete blood cell count (CBC) (Table 1) demonstrated anemia, with decreased RBC count, hematocrit, and hemoglobin relative to the Species360 Zoological Information Management System (ZIMS) reference intervals for Bengal tiger (*Panthera tigris tigris*).

Serum biochemical analysis (Table 2) revealed hyperproteinemia with hyperglobulinemia, mild hyperphosphatemia, and mild hypocholesterolemia, while renal indices (blood urea nitrogen [BUN] and creatinine) and hepatobiliary enzymes (alanine aminotransferase [ALT], alkaline phosphatase [ALP], and gamma-glutamyl transferase [GGT]) as well as total bilirubin were within the reported reference intervals. Overall, clinicopathologic abnormalities were consistent with anemia and inflammatory/chronic disease–associated changes that may accompany aggressive mammary carcinoma, extensive tissue injury, ulceration, and/or secondary infection.

For comprehensive diagnostic evaluation, general anesthesia was induced by intramuscular administration of ketamine (2 mg/kg) and medetomidine (0.025 mg/kg; Domitor) and maintained with inhalant anesthesia (isoflurane in oxygen at 2 L/min) delivered via endotracheal intubation and titrated to effect. Spontaneous ventilation was inadequate during induction, and assisted/mechanical ventilation was initiated immediately after induction and maintained throughout anesthesia (total 150 min). Peri-anesthetic monitoring included heart rate (65 beats/min), respiratory rate (10 breaths/min), capillary refill time (CRT; 1.5 s), and rectal temperature (37.5 °C). Contrast-enhanced computed tomography (CT) was performed using an institutional protocol. Post-contrast imaging was obtained following intravenous administration of a non-ionic iodinated contrast agent (iohexol; 300 mg I/mL) at 2 mL/kg (equivalent to 600 mg iodine/kg), selected as a weight-normalized dose consistent with our institutional veterinary CT protocol and within the range reported for veterinary contrast-enhanced CT [20]. CT acquisition and subsequent procedures (including biopsy performed in left lateral recumbency) were completed under assisted ventilation. During recovery, the animal did not regain sustained spontaneous respiration and failed to recover from anesthesia despite continued assisted ventilation and standard supportive measures; emergency cardiopulmonary resuscitation (CPR) was performed immediately.

**Figure 1:**
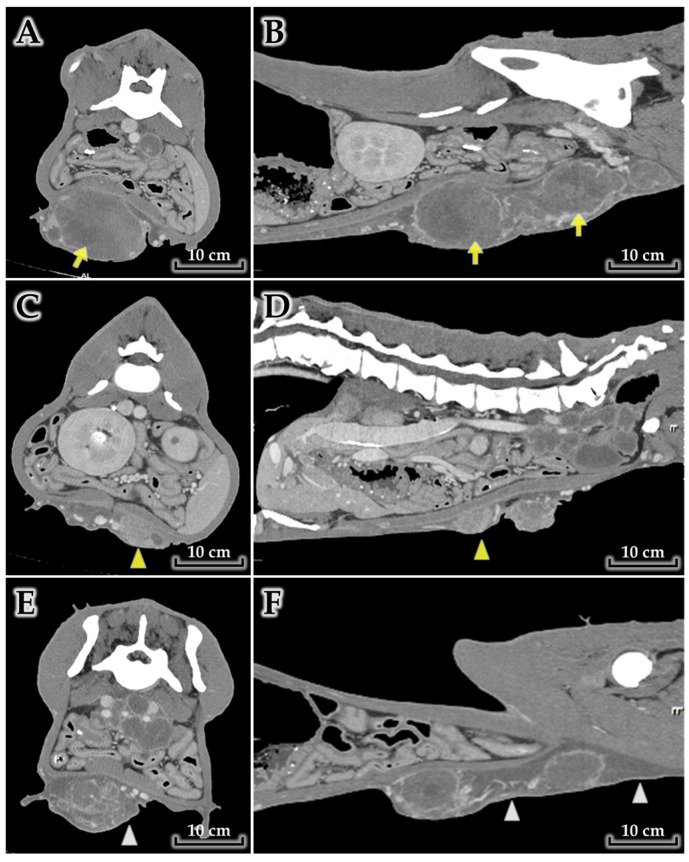
A mass extending from the right lower abdominal gland 4 to the posterior aspect (**A**,**B**) and the left mid-abdominal gland 3 (**C**,**D**), with adjacent inflammatory change (**E**,**F**). (**A**,**B**) Transverse (**A**) and sagittal (**B**) views of the abdomen showing a mammary mass measuring 15.6 × 12.2 × 45.0 cm (yellow arrow). (**C**,**D**) Transverse (**C**) and sagittal (**D**) views showing a second mammary mass measuring 8.8 × 4.2 × 8.1 cm (yellow arrowhead). (**E**,**F**) Transverse (**E**) and sagittal (**F**) views showing peritumoral inflammatory change with perilesional fluid attenuation (white arrowhead).

Contrast-enhanced CT demonstrated extensive mammary masses with adjacent inflammatory change (Figure 1). A very large mass centered in the right caudal abdominal mammary region (mammary gland 4) extended caudally and measured approximately 15.6 × 12.2 × 45.0 cm. The lesion showed heterogeneous enhancement with a peripheral rim-enhancing pattern and heterogeneous internal attenuation. A second mammary mass was identified in the left mid-abdominal mammary region (mammary gland 3), measuring approximately 9.8 × 4.2 × 8.1 cm, with similarly heterogeneous enhancement. The caudal aspect of the right-sided mass was accompanied by perilesional fluid attenuation and ill-defined adjacent soft tissue changes, interpreted as peritumoral inflammatory change/edema.

**Figure 2:**
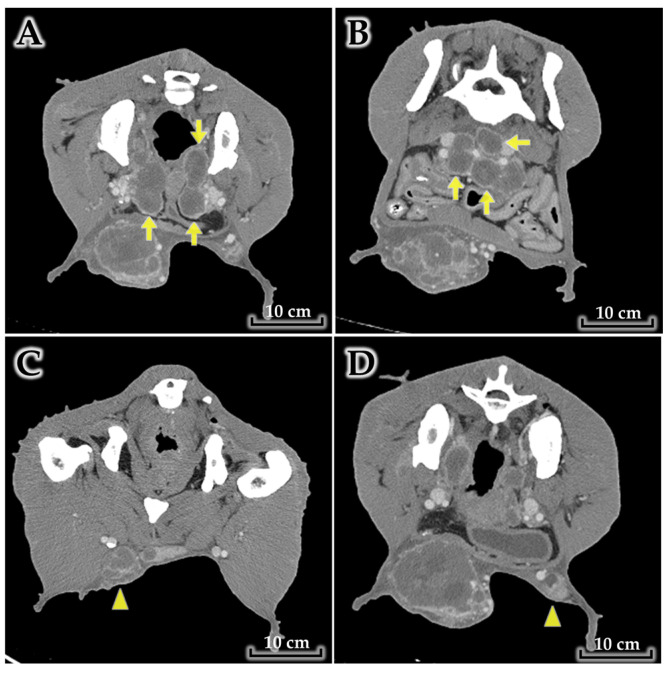
Regional lymphadenopathy on contrast-enhanced CT, suspicious for metastatic involvement. (**A**,**B**) Marked enlargement of the sublumbar (lumbar) lymph nodes (arrows) with structural alteration suggestive of neoplastic involvement. (**C**,**D**) Bilateral inguinal lymphadenopathy with features suspicious for regional metastasis (arrowheads indicate the inguinal lymph nodes).

Marked regional lymphadenopathy was present (Figure 2). The sublumbar (lumbar) lymph nodes were enlarged with structural changes suspicious for neoplastic involvement, and bilateral inguinal lymph nodes were also enlarged with features concerning for metastatic disease.

**Figure 3:**
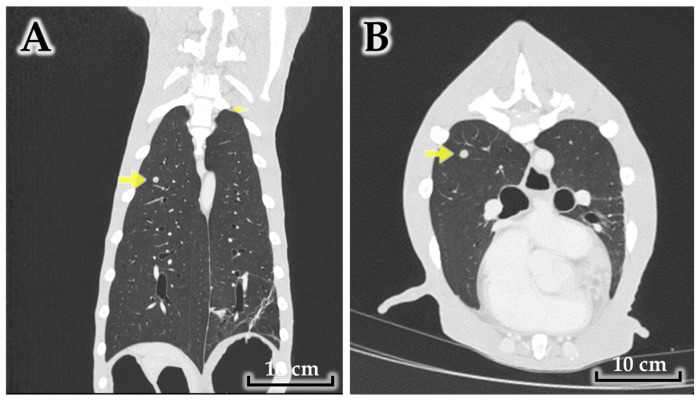
Small pulmonary nodule on thoracic CT. (**A**) Dorsoventral view showing a solitary 1 cm nodule in the right cranial lung lobe (arrow); (**B**) Transverse view of the same lesion (arrow). The lesion was suspicious for pulmonary metastasis in the clinical context of mammary carcinoma.

Thoracic imaging identified a discrete ~1 cm pulmonary nodule in the right cranial lung lobe (Figure 3), raising suspicion for pulmonary metastasis in the clinical context of mammary carcinoma.

Collectively, CT findings suggested multifocal mammary carcinoma with regional nodal involvement and suspected pulmonary metastasis, with prominent peritumoral inflammatory change compatible with an inflammatory tumor phenotype.

**Figure 4:**
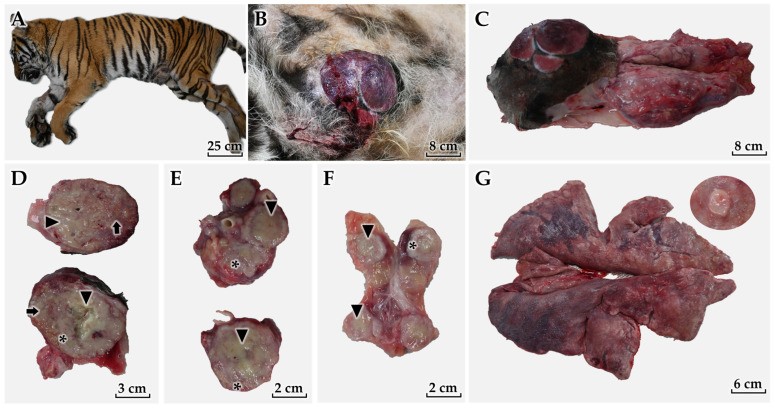
Necropsy and gross pathological findings. (**A**) External appearance at necropsy. (**B**) Large mammary mass (right gland 4) with severe cutaneous ulceration and hemorrhage. (**C**) Extensive coalescent mammary tumor mass extending caudally. (**D**) Cut surface of the primary tumor showing extensive central necrosis with suppurative change. (**E**) Markedly enlarged sublumbar lymph node with necrosis and inflammation. (**F**) Enlarged inguinal lymph node with gross changes suspicious for metastasis. (**G**) Lungs showing failure of collapse and gross changes suggestive of interstitial pneumonia; inset (upper right) shows a magnified view of an approximately 1 cm pulmonary nodule suspicious for metastasis. Figure S1 provides an in situ view to document tumor location and anatomic orientation. (**D**) and related gross panels show necrosis (arrows), suppurative exudate (arrow heads), and viable tumor tissue (asterisks).

The tiger failed to recover from anesthesia and subsequently died. A complete necropsy was performed immediately postmortem (Figure 4A). Given the suspected IMC phenotype—where assessment of the overlying skin and superficial dermis is diagnostically pivotal—tissue sampling included the mammary masses, regional lymph nodes (inguinal and sublumbar), lung (including the suspected metastatic nodule), as well as full-thickness skin containing superficial dermis and adjacent subcutis overlying and immediately adjacent to the mammary lesion, to enable direct evaluation of superficial dermal lymphatics for intralymphatic tumor emboli.

At necropsy, the right abdominal mammary gland (gland 4) contained a large mass with severe cutaneous ulceration and hemorrhage (Figure 4B), and an in situ photograph is provided for anatomic orientation (Figure S1). The mass extended markedly toward the caudal abdomen, forming an extensive coalescent tumor mass (Figure 4C). On the cut section, the primary mass exhibited extensive central necrosis with severe inflammation and purulent exudate, consistent with necrosuppurative change and likely secondary infection/abscessation within an aggressive neoplasm (Figure 4D); gross lesions are annotated on the images (arrows/asterisks) to indicate necrosis, purulent exudate, and viable tumor tissue.

Regional lymph nodes were markedly enlarged. The sublumbar lymph node showed prominent parenchymal disruption with necrosis and severe inflammatory change on cut surface (Figure 4E). The inguinal lymph node was also enlarged with hemorrhage, necrosis, and inflammatory changes consistent with metastatic involvement and secondary tissue injury (Figure 4F). The lungs failed to collapse and exhibited diffuse changes suggestive of interstitial pneumonia, and they contained a discrete nodule suspicious for metastasis (Figure 4G). Representative lung sections, including the pulmonary nodule and non-nodular parenchyma, were collected for histopathologic evaluation.

**Figure 5:**
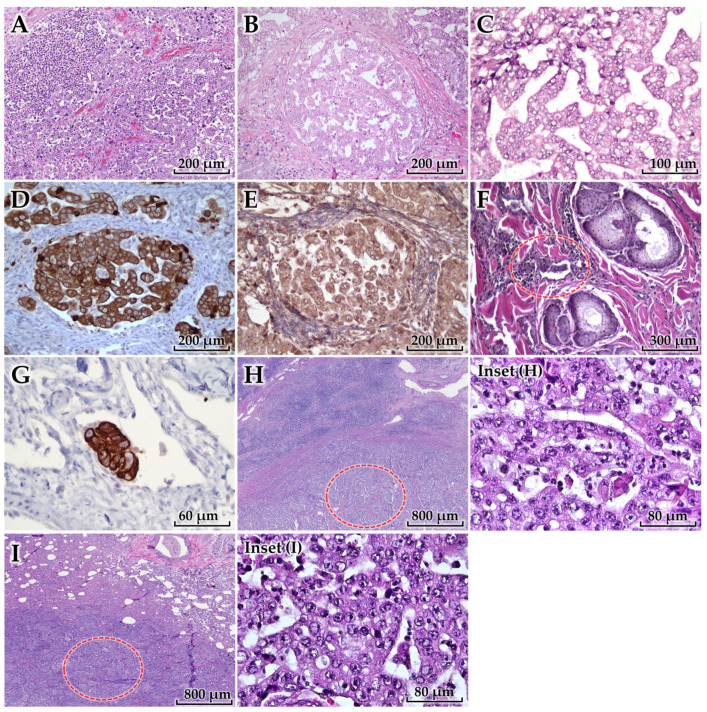
Histopathological and immunohistochemical findings of the mammary tumor and metastatic lesions. (**A**) Primary mammary carcinoma with marked tumor-associated inflammation extending into the superficial dermis (H&E). (**B**) Invasive carcinoma with a cribriform growth pattern and stromal desmoplasia (H&E). (**C**) High-grade cytologic atypia of neoplastic cells (H&E). (**D**,**E**) Neoplastic cells immunopositive for cytokeratin (**D**) and vimentin (**E**) (immunohistochemistry; [detection system], DAB chromogen, hematoxylin counterstain). (**F**) Tumor emboli within superficial dermal lymphatic vessels (red circle) (H&E). (**G**) Cytokeratin-positive dermal lymphatic tumor emboli (IHC; EnVision+ HRP polymer detection system (Agilent/Dako, Santa Clara, CA, USA), DAB chromogen, hematoxylin counterstain). (**H**) Metastatic carcinoma in the sublumbar lymph node (red circle) (H&E). (**I**) Pulmonary metastasis (red circle) (H&E). Scale bars are indicated in each subfigure. Higher-magnification (**Insets H**,**I**) are provided for (**H**,**I**).

Histologically, the primary mammary lesion was characterized by extensive infiltration of carcinoma cells accompanied by a severe diffuse inflammatory infiltrate composed predominantly of neutrophils with admixed lymphoid cells, extending from the subcutaneous tumor into the superficial dermis (Figure 5A). The tumor was composed of invasive nests of epithelial neoplastic cells forming multiple rounds and sharply demarcated lumina, creating a sieve-like cribriform architecture, with associated stromal desmoplasia (Figure 5B). Neoplastic cells exhibited marked anisocytosis and anisokaryosis, pronounced cellular and nuclear pleomorphism, and prominent nucleoli (Figure 5C).

Histologic grading was performed using a tubule formation/nuclear pleomorphism/mitotic count scoring system applied for feline mammary carcinoma [4], yielding a total score of 7 (Grade II malignancy) (tubule formation 3; nuclear pleomorphism 3; mitotic count 1). Mitotic figures were counted in 10 consecutive high-power fields (HPFs) in the most mitotically active viable peripheral tumor regions, avoiding necrotic/suppurative areas, and measured 8 mitoses/10 HPF, corresponding to a mitotic score of 1 using the thresholds defined by [4]. Immunohistochemistry demonstrated diffuse cytoplasmic immunoreactivity of neoplastic cells for cytokeratin (Figure 5D) and vimentin (Figure 5E), supporting epithelial lineage with co-expression of a mesenchymal marker compatible with phenotypic plasticity in an aggressive carcinoma.

Importantly, tumor emboli within dermal lymphatic vessels (dermal lymphatic invasion) were identified (Figure 5F; red circle). These intralymphatic tumor emboli exhibited strong cytokeratin immunoreactivity (Figure 5G), confirming epithelial tumor embolization within dermal lymphatics—a key pathologic feature supporting an IMC phenotype. Metastatic carcinoma was present within the sublumbar lymph node (Figure 5H; red circle), with higher-magnification insets provided to clearly demonstrate metastatic involvement, as well as metastatic foci in the lung (Figure 5I; red circle), with higher-magnification insets/additional panels to improve lesion visibility and publication clarity, confirming systemic dissemination.

Based on the combination of (i) an invasive (Grade II) mammary carcinoma with an invasive cribriform architecture and extensive lymphovascular invasion, (ii) tumor emboli within dermal lymphatic vessels, and (iii) metastatic carcinoma in regional lymph nodes and the lungs, the tumor was diagnosed as IMC.

## 3. Discussion

The present case describes an IMC-consistent mammary carcinoma in a captive Bengal tiger with extensive primary mammary disease, hallmark dermal lymphatic tumor emboli, and confirmed metastasis to regional lymph nodes and the lung. In managed-care nondomestic felids, where longevity and diagnostic scrutiny increase the detection of neoplasia, this case underscores the value of systematic necropsy, histopathology, and targeted submission of overlying full-thickness skin/subcutis to achieve a defensible diagnosis when an “inflammatory” phenotype is suspected [6,9].

A recurring challenge in IMC is that “inflammatory” is fundamentally a clinical descriptor rather than a purely histologic subtype; therefore, use of IMC terminology should be anchored on objective clinicopathologic criteria rather than secondary inflammatory changes alone. Secondary inflammation, ulceration, abscessation, or necrosis within advanced mammary carcinomas can mimic an inflammatory presentation and may confound interpretation. Accordingly, a robust diagnosis requires transparent integration of the clinical course with objective pathologic criteria—most notably, tumor emboli within superficial dermal lymphatic vessels (“dermal lymphatic tumor emboli”), which provide a morphologic correlate for the inflammatory clinical phenotype [12,14]. In the present case, intralymphatic tumor emboli were demonstrated within dermal lymphatics and confirmed by cytokeratin immunoreactivity, strengthening the clinicopathologic rationale for an IMC designation and minimizing ambiguity arising from prominent neutrophilic inflammation and ulceration.

The detection of dermal lymphatic tumor emboli is highly dependent on sampling strategy. If sampling is restricted to the deeper mammary mass, superficial dermal lymphatics may be underrepresented or absent, risking false-negative interpretation. For suspected IMC, full-thickness skin and subcutis overlying and immediately adjacent to the mammary lesion should be explicitly included in the sampling plan, along with careful step-sectioning and documentation of dermal lymphatic invasion [11,12]. In this report, the explicit inclusion of skin/subcutis enabled visualization of dermal lymphatic emboli and provided a reproducible basis for diagnosis. This point is particularly important for pathology reviewers, who commonly request image-based documentation of dermal lymphatic tumor emboli when IMC is claimed, and may recommend “inflammatory phenotype” terminology if this hallmark is not demonstrated or is inadequately documented [11,12]. Tumor-associated inflammation may be accompanied by immune dysregulation within the local tissue microenvironment, including altered functional states of CD4/CD8 T-cell populations [21].

Histologically, the primary tumor exhibited an invasive cribriform architecture with stromal desmoplasia, marked cytologic atypia, and extension into the superficial dermis. Although cribriform morphology is a recognized invasive growth pattern in feline mammary carcinoma [1,5], histologic pattern alone should not be over-interpreted as a surrogate for biologic aggressiveness, because its prognostic impact is variable across cohorts and is not consistently independent of other clinicopathologic factors [1]. In domestic cats, invasive mammary carcinomas encompass multiple growth patterns, and morphology is commonly interpreted alongside grade and invasion-/stage-related parameters (e.g., LVI and nodal status) for prognostic stratification [4,5,22]. While prognostic frameworks have been proposed and validated primarily for domestic cats, comparable species-specific validated prognostic systems are not currently available for tigers; therefore, applying felid-informed interpretive frameworks is most defensible when methods are transparent and limitations are explicitly acknowledged [9,22,23].

In the current case, histologic grade was assigned as Grade II based on tubule formation, nuclear pleomorphism, and mitotic count, while overall biologic behavior was unequivocally aggressive based on extensive lymphovascular invasion and confirmed systemic metastasis [4,5,22]. This apparent dissociation—intermediate grade but high metastatic propensity—has precedent in feline mammary carcinoma, where lymphovascular invasion and stage variables (including nodal involvement and distant metastasis) may outweigh grade alone in predicting outcome [4,5,22]. Accordingly, grading should be presented as one component of a pathology-based assessment, and the defining features supporting an inflammatory mammary carcinoma phenotype should be anchored by dermal lymphatic tumor emboli with rapid metastatic dissemination rather than by mitotic activity alone [11,12].

The immunophenotype in this case—diffuse cytokeratin positivity with concurrent vimentin expression—provides additional biologic context consistent with phenotypic plasticity. In feline mammary carcinoma, EMT-associated programs and basal-like phenotypes have been investigated using marker panels that include vimentin and basal cytokeratins, with several studies linking such patterns to invasiveness and metastatic competence [15,17,18]. Although vimentin expression in carcinoma cells is not specific for EMT and can reflect heterogeneous differentiation states, documenting CK/vimentin co-expression in a highly invasive tumor with marked lymphovascular invasion and metastasis is defensible and may support a narrative of enhanced migratory/invasive capacity [15]. This interpretation is based on the stains performed (cytokeratin and vimentin) and is integrated with morphology, invasion patterns, and clinicopathologic behavior.

From an epidemiologic and management perspective, mammary neoplasia is increasingly recognized in captive nondomestic felids, including *Panthera* spp., plausibly due to prolonged lifespan, enhanced clinical surveillance, and more frequent necropsy/histopathology compared with free-ranging counterparts [6,8,9]. Retrospective studies have also raised concern that exogenous progestin-based contraception (notably melengestrol acetate, MGA) may be associated with mammary carcinoma development and distinctive histologic features in zoo felids, underscoring the importance of documenting reproductive management history in mammary tumor investigations [10]. In the present case, inclusion of contraceptive exposure history (type, duration, cumulative dose, and timing relative to tumor detection) would improve interpretability and permit more meaningful comparison with prior managed-care felid series [10,23]. Likewise, age-related risk is relevant; neoplasia burden in captivity increases with age, and the advanced age of this tiger aligns with managed-care patterns described in *Panthera* neoplasia series [9].

Several limitations should be acknowledged. First, grading systems and prognostic thresholds have not been validated for tigers, and any use of domestic-cat-informed grading must be presented as an interpretive tool rather than a species-specific predictor [4,17]. Second, because IMC is clinicopathologic by nature, the diagnostic argument is strongest when the rapid inflammatory clinical course and the dermal lymphatic emboli are both comprehensively documented; if any component is incomplete, cautious terminology such as “mammary carcinoma with an inflammatory phenotype” may be more conservative [11,12]. Third, the grossly necrosuppurative character of the mass raises the possibility of secondary bacterial infection, which can intensify local inflammation and complicate clinical interpretation; bacteriologic culture and/or ancillary testing of purulent exudate would help clarify the contribution of secondary infection to the clinical syndrome [24]. In addition, systemic hyperinflammatory syndromes characterized by immune hyperactivation and dysregulation (e.g., hemophagocytic lymphohistiocytosis) may be precipitated by infection or malignancy and can further confound clinical assessment in severely inflamed patients [25]. In this context, the antemortem anemia and systemic inflammatory abnormalities are not specific to neoplasia and may also accompany concurrent systemic inflammatory/immune activation syndromes; therefore, non-neoplastic contributors cannot be fully excluded without targeted ancillary testing [26]. Finally, the lungs displayed gross features suggestive of interstitial pneumonia; histologically, non-neoplastic lung parenchyma showed moderate diffuse interstitial (alveolar septal) thickening with mixed inflammatory cell infiltration (neutrophils and lymphocytes), accompanied by multifocal edema and congestion, consistent with interstitial pneumonia, in addition to the metastatic focus described above. No special stains or ancillary infectious testing were performed; therefore, the etiology (infectious vs. noninfectious) could not be determined. Such pulmonary compromise may have reduced respiratory reserve and potentially increased anesthetic risk, although a causal relationship cannot be established in this case [27].

In conclusion, this case expands the documented spectrum of aggressive mammary carcinoma phenotypes in managed-care nondomestic felids by providing clinicopathologic evidence consistent with IMC in a captive Bengal tiger. The presence of cytokeratin-confirmed dermal lymphatic tumor emboli, together with extensive regional and distant metastasis, supports an IMC designation and highlights the diagnostic necessity of intentional skin/subcutis sampling when an inflammatory phenotype is suspected. Systematic pathology-centered investigation, transparent grading methodology, and immunophenotypic characterization can strengthen interpretive rigor and facilitate meaningful comparison across domestic and nondomestic felid mammary carcinoma reports.

## 4. Conclusions

This case documents inflammatory mammary carcinoma in a captive Bengal tiger, supported by cytokeratin-confirmed dermal lymphatic tumor emboli and metastases to regional lymph nodes and the lung. These findings broaden clinicopathologic recognition of IMC in managed-care nondomestic felids and reinforce that diagnostic confirmation depends on intentional full-thickness skin/subcutis sampling to capture superficial dermal lymphatics. As a single-case report, species-specific prognostic inference is limited, and additional multicenter case accrual using standardized diagnostic workflows is needed. Future investigations integrating uniform sampling, imaging, and ancillary testing will strengthen diagnostic consistency and improve cross-institutional comparability and surveillance of aggressive mammary carcinoma phenotypes in captive felids.

## Figures and Tables

**Table 1:** Complete blood cell count (CBC) results.

Parameter	Result	Unit	Reference Interval (ZIMS *)	Parameter	Result	Unit	Reference Interval (ZIMS)
RBC	4.66	M/μL	4.73–8.88	%LYM	13.9	%	0.0–33.0
HCT	22.3	%	28.4–51.1	%MONO	7.4	%	0.0–7.3
HGB	7.3	g/dL	9.2–16.8	%EOS	0	%	0.0–7.0
MCV	47.9	fL	46.8–64.3	%BASO	0.3	%	0.0–1.0
MCH	15.7	pg	16.8–22.3	NEU	8.43	K/μL	3.84–17.510
MCHC	32.7	g/dL	28.4–37.3	LYM	1.49	K/μL	0.662–4.207
RDW	19.6	%	17.8–25.1	MONO	0.79	K/μL	0.091–0.896
WBC	10.74	K/μL	5.8–22.2	EOS	0	K/μL	0.049–0.820
%NEU	78.4	%	48.8–88.0	PLT	242	K/μL	109–420

* Reference intervals were obtained from the Species360 Zoological Information Management System (ZIMS) medical reference intervals database (Species360, Bloomington, MN, USA; 2017).

**Table 2:** Serum biochemical profile.

Parameter	Result	Unit	Reference Interval (ZIMS *)	Parameter	Result	Unit	Reference Interval (ZIMS)
Na^+^	147.7	mmol/L	144–159	TP	9.7	g/dL	5.7–8.8
K^+^	4.28	mmol/L	3.4–5.1	GLOB	7.1	g/dL	2.2–5.9
Cl^−^	117.1	mmol/L	113–128	ALT	40	U/L	24–115
GLU	111	mg/dL	61–192	ALKP	<10	U/L	7–47
CREA	2	mg/dL	0.8–4.1	GGT	0	U/L	0–8
BUN	34	mg/dL	16.0–48.1	TBIL	<0.1	mg/dL	0.1–0.6
PHOS	8.9	mg/dL	3.8–8.4	CHOL	144	mg/dL	146–320
Ca	9.2	mg/dL	8.6–11.2	AMYL	2178	U/L	64–3926
				BUN/CREA	17		5.3–30.0

* Reference intervals were obtained from ZIMS Species360.

## Data Availability

All data are contained within the article.

## Supplementary Materials

The following supporting information can be downloaded at: https://www.mdpi.com/article/10.3390/ani16050757/s1, Figure S1: In situ gross appearance of the right caudal abdominal mammary mass (mammary gland 4) prior to excision, showing its anatomic relationship to the mammary chain and adjacent ventral abdominal tissues.
