# Supplementary material for: Inflammatory Mammary Carcinoma in a Captive Bengal Tiger (Panthera tigris tigris) with Lymph Node and Pulmonary Metastases
Source: Animals (Basel). 2026 Mar 1;16(5):757. doi: 10.3390/ani16050757 (PMC12985059; doi:10.3390/ani16050757)
Supplement: Supplementary file 1 [file animals-16-00757-s001.zip › animals-4094366-supplementary.pdf]

Supplementary Materials

Supplementary Figure

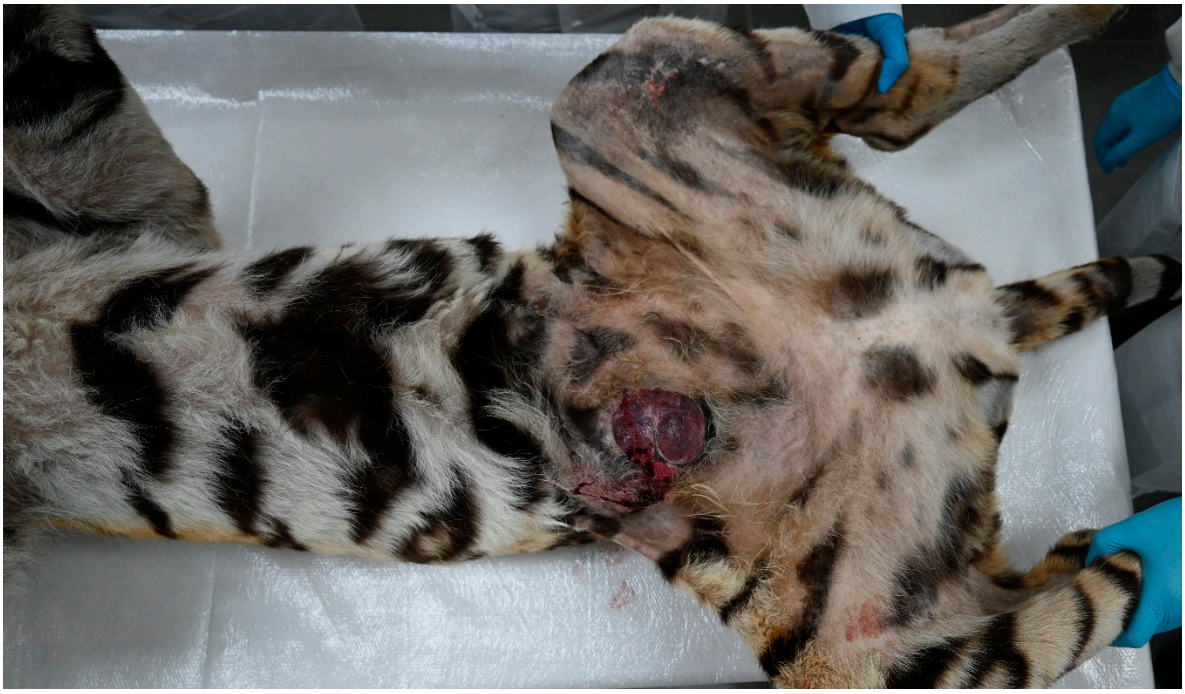

Supplementary Figure S1. In situ gross appearance of the right caudal abdominal mammary mass (mammary gland 4) prior to excision, showing its anatomic relationship to the mammary chain and adjacent ventral abdominal tissues.
